# Accelerated passage of gene-modified monkeys by hormone-induced precocious puberty

**DOI:** 10.1093/nsr/nwab083

**Published:** 2021-05-04

**Authors:** Zhen Liu, Kui Li, Yijun Cai, Qiming Liu, Tikui Zhang, Yong Lu, Yanhong Nie, Yan Wang, Zhiguang Yan, Yinwei Qu, Yuzhuo Li, Zhanyang Wang, Zhi-Qi Xiong, Qiang Sun

**Affiliations:** Institute of Neuroscience, State Key Laboratory of Neuroscience, CAS Key Laboratory of Primate Neurobiology, CAS Center for Excellence in Brain Science and Intelligence Technology, Chinese Academy of Sciences, Shanghai Research Center for Brain Science and Brain-Inspired Intelligence, China; Institute of Neuroscience, State Key Laboratory of Neuroscience, CAS Key Laboratory of Primate Neurobiology, CAS Center for Excellence in Brain Science and Intelligence Technology, Chinese Academy of Sciences, Shanghai Research Center for Brain Science and Brain-Inspired Intelligence, China; Institute of Neuroscience, State Key Laboratory of Neuroscience, CAS Key Laboratory of Primate Neurobiology, CAS Center for Excellence in Brain Science and Intelligence Technology, Chinese Academy of Sciences, Shanghai Research Center for Brain Science and Brain-Inspired Intelligence, China; Institute of Neuroscience, State Key Laboratory of Neuroscience, CAS Key Laboratory of Primate Neurobiology, CAS Center for Excellence in Brain Science and Intelligence Technology, Chinese Academy of Sciences, Shanghai Research Center for Brain Science and Brain-Inspired Intelligence, China; Institute of Neuroscience, State Key Laboratory of Neuroscience, CAS Key Laboratory of Primate Neurobiology, CAS Center for Excellence in Brain Science and Intelligence Technology, Chinese Academy of Sciences, Shanghai Research Center for Brain Science and Brain-Inspired Intelligence, China; Institute of Neuroscience, State Key Laboratory of Neuroscience, CAS Key Laboratory of Primate Neurobiology, CAS Center for Excellence in Brain Science and Intelligence Technology, Chinese Academy of Sciences, Shanghai Research Center for Brain Science and Brain-Inspired Intelligence, China; Institute of Neuroscience, State Key Laboratory of Neuroscience, CAS Key Laboratory of Primate Neurobiology, CAS Center for Excellence in Brain Science and Intelligence Technology, Chinese Academy of Sciences, Shanghai Research Center for Brain Science and Brain-Inspired Intelligence, China; Institute of Neuroscience, State Key Laboratory of Neuroscience, CAS Key Laboratory of Primate Neurobiology, CAS Center for Excellence in Brain Science and Intelligence Technology, Chinese Academy of Sciences, Shanghai Research Center for Brain Science and Brain-Inspired Intelligence, China; Department of Assisted Reproduction, Shanghai Ninth People's Hospital, Shanghai Jiaotong University School of Medicine, China; Institute of Neuroscience, State Key Laboratory of Neuroscience, CAS Key Laboratory of Primate Neurobiology, CAS Center for Excellence in Brain Science and Intelligence Technology, Chinese Academy of Sciences, Shanghai Research Center for Brain Science and Brain-Inspired Intelligence, China; Institute of Neuroscience, State Key Laboratory of Neuroscience, CAS Key Laboratory of Primate Neurobiology, CAS Center for Excellence in Brain Science and Intelligence Technology, Chinese Academy of Sciences, Shanghai Research Center for Brain Science and Brain-Inspired Intelligence, China; Institute of Neuroscience, State Key Laboratory of Neuroscience, CAS Key Laboratory of Primate Neurobiology, CAS Center for Excellence in Brain Science and Intelligence Technology, Chinese Academy of Sciences, Shanghai Research Center for Brain Science and Brain-Inspired Intelligence, China; Institute of Neuroscience, State Key Laboratory of Neuroscience, CAS Key Laboratory of Primate Neurobiology, CAS Center for Excellence in Brain Science and Intelligence Technology, Chinese Academy of Sciences, Shanghai Research Center for Brain Science and Brain-Inspired Intelligence, China; Institute of Neuroscience, State Key Laboratory of Neuroscience, CAS Key Laboratory of Primate Neurobiology, CAS Center for Excellence in Brain Science and Intelligence Technology, Chinese Academy of Sciences, Shanghai Research Center for Brain Science and Brain-Inspired Intelligence, China

**Keywords:** non-human primates, gene editing, passage acceleration, precocious puberty

Mosaicism occurs in most founder gene-modified monkeys generated by virus infection and programmable-nucleases [1–3]. In addition, macaque monkeys have a long preadolescence period, with four or more years usually required for male macaque monkeys to become sexually mature [4,5]. The mosaicism and long time to sexual maturity have largely prevented wide usage of gene-modified monkey models.

Accelerating F1 generation could be significant in development of animal models using gene-modified monkeys. In previous studies focused on hormonal control of sexual development and neuroendocrine mechanism of puberty onset, multiple exogenous hormones and drugs showed puberty-augmenting effects to varying degrees in juvenile male monkeys [6–8]. However, no detailed study has focused on obtaining functional sperm and accelerating offspring generation by exogenous factors in precocious male monkeys.

Here, we describe acceleration of monkey passage by precocious puberty induction of juvenile male monkeys. We first investigated the normal sexually mature age (motile sperm generation) of male monkeys in our colony (Suzhou, China). We collected semen samples from 37 male monkeys: 8 monkeys aged 36–42 months, 7 monkeys aged 45–48 months, 17 monkeys aged 50–55 months and 5 monkeys aged 59–66 months. There were no sperm in the samples from any of the 15 monkeys aged 36–42 months and 45–48 months; however, 13/17 monkeys in the 50–55 months group and 4/5 monkeys in 59–66 months group generated motile sperm in their semen (Fig. 1A). This indicates that age 50 months is the threshold for sexual maturity in the male cynomolgus monkeys in our colony (Fig. 1B).

To accelerate the process of sexual maturity, nine juvenile male monkeys (*Macaca fascicularis*) in three age groups (∼0.5 year, n = 2; ∼1 year, n = 4; ∼2 year, n = 3) were treated with intramuscular injections of human follicle stimulating hormone (FSH, 3 IU/kg/day) and testosterone (testosterone enanthate, 125 mg/week). The hormone doses used in this study were similar to those from previous studies [7]. Three untreated monkeys (∼1 year old) were used as control. Monkey semen was collected by penile electro-ejaculation and checked for the presence of sperm under an inverted microscope. After 4–6 months of hormone injection, motile sperm were detected in semen from 4/9 treated monkeys (Fig. 1B and C). No sperm were generated by the three untreated control monkeys. The four monkeys that generated mature sperm were from the 1-year (n = 2, 2/4) and 2-year (n = 2, 2/3) groups, with no sperm appearing in samples from the two monkeys in the 0.5-year group (n = 2, 0/2) (Supplementary Table S1).

The success rate in obtaining viable sperm was limited (4/9) in the first-round experiment, so it was thought that extending the hormone treatment time might improve this. In our second-round experiment, five juvenile male monkeys (*Macaca fascicularis*) of two age groups (∼0.5 year, n = 2; ∼1 year, n = 3) were treated following the same method as in the first-round experiment. Another three untreated monkeys (∼1 year old) were used as the control. Motile sperm were obtained from all three 1-year-old treated juvenile male monkeys after hormone treatment for 7, 11 and 11 months, respectively. Motile sperm were obtained from both 0.5-year-old monkeys after hormone treatment for 8 and 11 months, respectively. No sperm were generated by the three untreated control moneys. Overall, we succeeded in collecting sperm from all five hormone-treated monkeys by extending the hormone treatment time (Fig. 1D and Supplementary Table S1).

Physical examination of the monkeys was performed monthly to record body weight in the two rounds of experiments and the testicular volume in the second-round experiment. Compared with the control monkeys, the mean body weight and the testicular volume of hormone-treated monkey groups increased significantly over the course of the hormone treatment (Fig. 1E, F and G). Hematoxylin-eosin staining was performed on testicular tissue sections from the hormone-treated monkeys producing sperm and age-matched control monkeys. The samples showed the presence of mature sperm in the treated monkeys compared with the immature testicular tissue of the age-matched control monkeys (Fig. 1H and I).

**Figure 1.**
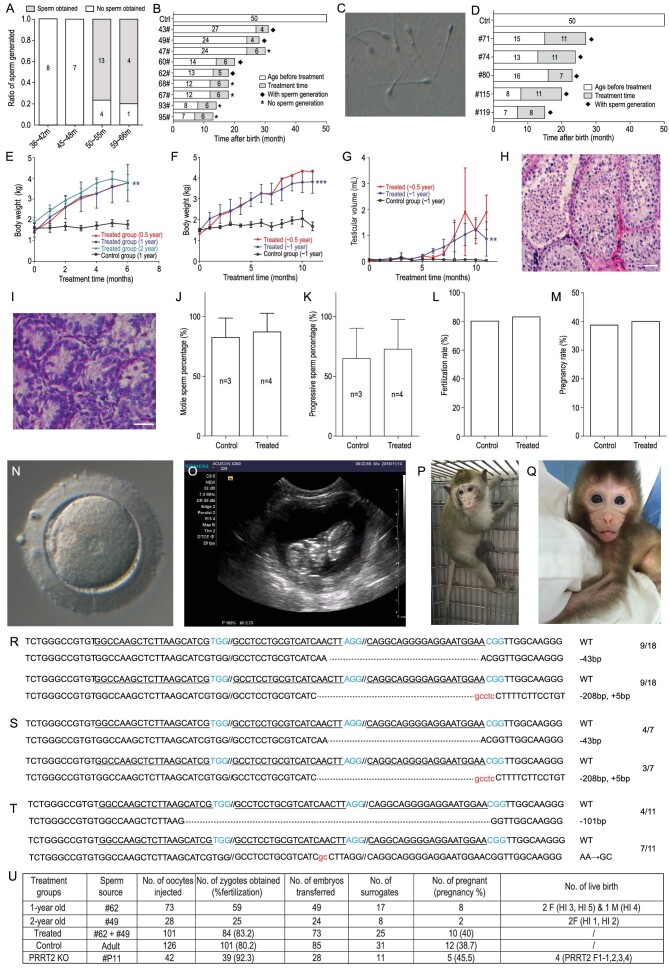
High efficiency of sperm and offspring generation from precocious juvenile male monkeys by FSH and testosterone treatment. (A) Motile sperm generation in male macaque monkeys of different ages from a Suzhou colony. (B) A list of motile sperm generation in first round hormone-treated WT monkeys, the control indicating the time for sperm natural maturation. (C) A microscopic photo of sperm derived from ejaculated semen of #62 precocious monkeys. (D) 100% success rate of motile sperm generation in second round hormone-treated WT monkeys, the control indicating the time for sperm natural maturation. (E) Growth curves of body weight of first round hormone-treated monkeys and control monkeys. (F) Growth curves of body weight of second round hormone-treated monkeys and control monkeys. (G) Testicular volume comparisons between the hormone-treated monkeys and control monkeys in the second round experiment. (H) Sections of testicular tissue from monkey #62 (23 months) show sperm appearance in the precocious monkey (bar = 20 μm). Arrows 1–5 indicate round spermatid, elongated spermatid, pathytene spermatocyte, dark spermatogonial stem cell, and pale spermatogonial stem cell. (I) Sections of testicular tissue from natural control monkeys of similar age (26 months) to monkey #62 (bar = 20 μm). (J and K) Motile and progressive rate comparison between sperm from natural and precocious monkeys. (L) Fertilization rate comparison between sperm from natural and precocious monkeys. (M) Pregnancy rate comparison between embryos constructed using sperm from natural and precocious monkeys. (N) A zygote with two pronuclei constructed using sperm from precocious monkeys. (O) An ultrasound image from a pregnant monkey carrying an HI monkey. (P) Precocity monkey #62 (sperm appeared at 18 months old and picture taken at 25 months old). (Q) Monkey HI 5 derived using sperm from monkey #62. (R) Genotype analysis of monkey embryos generated using sperm from PRRT2 F0 founder P11. (S) Genotype analysis of monkey offspring generated using sperm from PRRT2 F0 founder P11. (T) Genotype analysis of monkey embryos generated using sperm from PRRT2 F0 founder P12. (U) Efficiency of generating F1 monkeys.

Motility and quality of the sperm from four hormone-treated monkeys and three naturally matured (5-year-old male) control monkeys were tested by computer-aided sperm analysis (CASA), which was developed for human semen quality analysis [9]. For the three control monkeys, the rates of motile sperm were 94.7%, 88.7% and 64%. The corresponding rates of progressive sperm (grade ‘A’ and ‘B’) were 86.8%, 70.9% and 37.2%. For the four hormone-treated monkeys, the rates of motile sperm were 97.4%, 95.2%, 91.8% and 63.9%. The corresponding rates of progressive sperm were 90.9%, 86.3%, 77.6% and 36.4%. Both the control and hormone-treated groups included a monkey whose sperm showed low motility and quality. Meanwhile the rates of motile sperm and the rates of progressive sperm showed no obvious differences between the control samples and the hormone-treated samples, indicating similar quality in the sperm from the hormone treated monkeys and the naturally matured control monkeys (Fig. 1J and K).

To evaluate the epigenetic status in sperm from the precocious monkeys, whole genome and single-base resolution maps of 5-methylcytosine (mC) analysis were performed by bisulfite-sequencing (BS-Seq) at an average depth of 15.4-fold per strand in a DNA sample from the sperm of a naturally matured monkey (#2) and 13.6-fold in a sample from a sexually precocious monkey (#49). Around 70% of all cytosine in the genome was covered at least five times in each sample. We analyzed the average methylation levels and density of cytosine in the two samples and found no significant difference between the hormone-treated group and the control group (Supplementary Fig. S1). We further analyzed the DNA methylation level of imprinted genes ATP10A, UBE3A, DLK1, PLAGL1, PEG3 and IGF2R, and found similar methylation levels in the region (16–180 kb) of these genes from the two sperm samples (Supplementary Fig. S2).

Sperm from two hormone-treated monkeys were injected into monkey MII-stage oocytes by ICSI and the resultant embryos were transferred to synchronized surrogates. For 2-year old treatment group, sperm from monkey #49 (after 4-month hormone treatment) were used for ICSI. Twenty-eight oocytes were injected and 25 zygotes were obtained, among which 24 transferred to eight surrogate monkeys, resulting in two pregnancies that both yielded healthy offspring (named HI 1 and HI 2; HI, ‘hormone injection’). For 1-year old treatment group, sperm from monkey #62 (after 5-month hormone treatment) was used in ICSI on 73 oocytes and 59 zygotes were obtained, among which 49 transferred to 17 surrogate monkeys, resulting in eight pregnancies that yielded three healthy infant monkeys (named HI 3, HI 4, HI 5, Fig. 1N–Q), with the rest either miscarried or stillborn. The main reason for miscarriage may be caused by twin and triplet pregnancy (two twins, one triplet, one singlet). In total, 101 oocytes were used for ICSI and 84 zygotes were obtained using sperm from a hormone-treated juvenile monkey. The fertility rate was 83.2% and showed no difference compared with that using naturally matured sperm. The pregnancy rate was 40% (10/25), also similar to that using naturally matured sperm (Fig. 1L and M). Taken together, we have generated 10 pregnant surrogates and five healthy monkey offspring by ICSI using sperm derived from two hormone-treated juvenile monkeys of different age groups (Fig. 1U).

Short tandem repeat (STR) genotyping was performed to examine the parental origin of the five live HI monkeys. Analysis of 24 STR loci of the five live F1 offspring monkeys confirmed their paternal origin (Supplementary Table S2). Physical examination was conducted in all five HI monkey offspring derived from the sperm of juvenile monkeys. The body weight, abdominal and head circumferences, head-truck length, heart and respiratory rates and body temperature were recorded weekly after the monkeys were born. No significant differences were found in four physical parameters (body weight, abdominal and head circumferences and head-truck length) between five HI monkeys and six age-matched control monkeys during 23 months (Supplementary Fig. S3). No detectable abnormality was found in other observed traits. Thus, the sperm from the hormone-treated juvenile monkeys have the same quality in terms of generating healthy offspring as the sperm from naturally matured monkeys.

Next, we applied this hormone-induced sexual mature method in shortening the passage of gene-modified monkeys. Previously, we reported the generation of PRRT2 knock out monkeys [10]. To accelerate the F1 monkey generation, two 12-month-old PRRT2 knock out F0 monkeys (named P11 and P12) were used for hormone treatment. After 7 months and 12 months of hormone injections, we succeeded in obtaining motile sperm from P11 and P12, and embryos were constructed by ICSI using this sperm. Genotype analysis of embryos cultured to blastocyst stage *in vitro* was performed by PCR and DNA sequencing. The results showed that all the sequenced embryos were monogenic mutant in the target sequence of PRRT2 gene (Fig. 1R, T). Thirty-nine zygotes were obtained using 42 oocytes generated with P11 sperm, 28 PRRT2 F1 embryos were transferred to 11 surrogates, five pregnant surrogates with seven fetuses were confirmed and four live birth PRRT2 F1 offspring were obtained (Supplementary Fig. S4). Genotype analysis showed that all seven live fetuses were monogenic mutant in the target sequence of PRRT2 gene as detected previously for the embryos (Fig. 1S and Supplementary Table S3).

The hormone-induced precocious method was also applied to accelerate passage of MECP2-GFP transgenic monkeys [2]. A 36-month-old F0 MECP2-GFP transgenic monkey (named M08) was hormone-treated and motile sperm appeared 4 months after induction. Three live MECP2-GFP F1 transgenic offspring were obtained using sperm from M08. The presence of transgene GFP was confirmed by PCR analysis in the three live F1 offspring (named M08F1-1, M08F1-2, M08F1-3). When M08F1-1 grew to 12 months old, we applied hormone treatment to this male MECP-GFP F1 transgenic monkey. After 8 months of treatment, motile sperm appeared in the semen of M08F1-1. Three MECP-GFP F2 transgenic monkeys (named M08F2-1, M08F2-2, M08F2-3) were generated using sperm from M08F1-1. The presence of transgene GFP was confirmed by PCR analysis in the MECP-GFP F2 monkeys (Supplementary Fig. S5).

In summary, we have largely shortened the generation time of gene-modified F1 and F2 monkey offspring by precocious induction using testosterone and FSH injection. We believe that this could be used as a common and easy method to accelerate passage of gene-modified monkey models.

## Supplementary Material

nwab083_Supplemental_Files

## Data Availability

The accession number of the WGBS reported in this study is GEO: GSE167929.

## STATISTICAL ANALYSIS

Differences of gene expression levels between groups were analyzed by Student's *t* test.
