# Supplementary material for: Accelerated passage of gene-modified monkeys by hormone-induced precocious puberty
Source: Natl Sci Rev. 2021 May 4;8(7):nwab083. doi: 10.1093/nsr/nwab083 (PMC8310752; doi:10.1093/nsr/nwab083)
Supplement: nwab083_Supplemental_Files [file nwab083_supplemental_files.zip › supplementary_figures_and_tables_list.docx]

**Supplementary Figure S1. Epigenome analysis of sperm from the precocious monkey and control monkey. (A, B)** Methylation level of total C (cytosine) and CpG island in each individual chromosome of sperm from the two samples. **(C, D)** Methylation density in each individual chromosome of sperm from the two samples. **(E, F)** The distribution of sites with different methylation level and density across whole genome in two sample.

**Supplementary Figure S2. DNA methylation analysis of imprinted gene regions in sperm samples of precocious monkey and control monkey**

**.**

**Supplementary Figure S3. Growth curves based on body weight, head circumference, head-truck length and abdominal circumference in HI monkeys and control monkeys.**

**Supplementary Figure S4**. **Photos of four heterozygous PRRT2 knock-out monkey offspring generated using sperm from P11.**

**Supplementary Figure S5. Photos of three MECP2-GFP F2 transgenic monkey offspring and the genotype analysis of MECP2-GFP transgenic monkeys.**

**Supplementary Table S1. Sperm generation in hormone-treated and control monkeys.**

**Supplementary Table S2. STR analysis of HI monkeys.**

**Supplementary Table S3. Summary of PRRT2 F1 monkeys**
