# Supplementary material for: Accelerated passage of gene-modified monkeys by hormone-induced precocious puberty
Source: Natl Sci Rev. 2021 May 4;8(7):nwab083. doi: 10.1093/nsr/nwab083 (PMC8310752; doi:10.1093/nsr/nwab083)
Supplement: nwab083_Supplemental_Files [file nwab083_supplemental_files.zip › Supplementary_Table_S1.docx]

| **Groups (monkey #)** | | **Monkey Number** | **Age before treatment (months)** | **Treatment time (months)** | **Sperm obtained**  **(monkey age in months)** |
| --- | --- | --- | --- | --- | --- |
| **1 round**  **experiment** | **0.5-year old +**  **treatment (2)** | 093 # | 8 | 6 | No |
|  |  | 095 # | 7 | 6 | No |
|  | **1- year old +**  **treatment (4)** | 060 # | 14 | 6 | Yes (20) |
|  |  | 062# | 13 | 5 | Yes (18) |
|  |  | 067 # | 12 | 6 | No |
|  |  | 068# | 12 | 6 | No |
|  | **2-year old +**  **treatment (3)** | 043 # | 27 | 4 | Yes (31) |
|  |  | 047# | 24 | 6 | No |
|  |  | 049 # | 24 | 4 | Yes (28) |
|  | **1-year old+**  **control (3)** | 078# | 15 | / | No |
|  |  | 079# | 15 | / | No |
|  |  | 081# | 15 | / | No |
| **2 round**  **experiment** | **0.5-year old +**  **treatment (2)** | 0115# | 8 | 11 | Yes (19) |
|  |  | 0119# | 7 | 8 | Yes (15) |
|  | **1- year old +**  **treatment (3)** | 071# | 15 | 11 | Yes (27) |
|  |  | 074# | 13 | 11 | Yes (24) |
|  |  | 080# | 16 | 7 | Yes (23) |
|  | **1-year old+**  **control (3)** | 065# | 16 | / | No |
|  |  | 072# | 14 | / | No |
|  |  | 082# | 16 | / | No |
| **3 round**  **experiment** | **Prrt2 KO (2)** | P11 | 12 | 12 | Yes (24) |
|  |  | P12 | 12 | 7 | Yes (19) |

**Supplementary Table S1. Sperm generation in Hormone-treated and Control Monkeys**
