# Supplementary material for: Accelerated passage of gene-modified monkeys by hormone-induced precocious puberty
Source: Natl Sci Rev. 2021 May 4;8(7):nwab083. doi: 10.1093/nsr/nwab083 (PMC8310752; doi:10.1093/nsr/nwab083)
Supplement: nwab083_Supplemental_Files [file nwab083_supplemental_files.zip › Supplementary_Table_S2.docx]

**Supplementary Table S2, STR analysis of HI monkeys**

| **Locus** | **Sperm origin** | **Oocyte origin** | **HI monkeys** | |
| --- | --- | --- | --- | --- |
|  | **#49** | **#282** | **HI 1** | **HI 2** |
| **D1S548** | **188/196** | **196/200** | **188/196** | **196/200** |
| **D2S1333** | **275/335** | **290/297** | **275/290** | **297/335** |
| **D3S1768** | **183/233** | **212/220** | **183/220** | **212/233** |
| **D4S2365** | **283/287** | **280/280** | **280/283** | **280/287** |
| **D4S413** | **131/135** | **133/135** | **133/135** | **135/135** |
| **D5S1457** | **124/128** | **116/124** | **116/124** | **124/124** |
| **D6S291** | **211/217** | **205/209** | **205/211** | **209/211** |
| **D6S1691** | **202/210** | **192/198** | **192/202** | **198/202** |
| **D6S501** | **167/175** | **167/179** | **167/179** | **167/175** |
| **D7S513** | **195/204** | **191/201** | **195/201** | **191/204** |
| **D7S794** | **145/154** | **150/154** | **154/154** | **154/154** |
| **D8S1106** | **215/218** | **206/218** | **206/218** | **206/218** |
| **D9S921** | **174/178** | **170/186** | **170/178** | **170/174** |
| **D10S1412** | **161/161** | **161/164** | **161/161** | **161/164** |
| **D11S2002** | **226/238** | **238/238** | **226/238** | **226/238** |
| **D12S364** | **267/278** | **256/257** | **257/278** | **257/278** |
| **D13S765** | **225/230** | **225/225** | **225/230** | **225/230** |
| **D15S823** | **381/389** | **325/329** | **325/381** | **329/389** |
| **D18S72** | **320/320** | **326/328** | **320/328** | **320/328** |
| **D18S537** | **174/174** | **174/174** | **174/174** | **174/174** |
| **D22S685** | **351/351** | **343/351** | **351/351** | **351/351** |
| **DXS2506** | **259/270** | **266/270** | **266/270** | **266/270** |
| **MFGT21** | **110/116** | **118/120** | **116/120** | **110/120** |
| **MFGT22** | **98/106** | **110/116** | **106/110** | **106/110** |

| **Locus** | **Sperm origin** | **Oocyte origin** | | | **HI monkeys** | | |
| --- | --- | --- | --- | --- | --- | --- | --- |
|  | **#62** | **#332 (HI 3)** | **#400 (HI 4)** | **#423 (HI 5)** | **HI 3** | **HI 4** | **HI 5** |
| **D1S548** | **188/196** | **192/196** | **192/196** | **192/200** | **196/196** | **192/196** | **188/192** |
| **D2S1333** | **273/284** | **281/308** | **296/296** | **285/300** | **273/308** | **284/296** | **273/300** |
| **D3S1768** | **183/187** | **199/224** | **204/212** | **199/220** | **183/199** | **183/204** | **187/220** |
| **D4S2365** | **283/287** | **279/283** | **268/283** | **283/283** | **283/287** | **268/287** | **283/287** |
| **D4S413** | **132/134** | **123/130** | **130/132** | **128/136** | **130/134** | **132/134** | **128/134** |
| **D5S1457** | **124/128** | **124/128** | **120/120** | **124/124** | **128/128** | **120/128** | **124/128** |
| **D6S291** | **210/216** | **204/210** | **208/210** | **208/208** | **204/210** | **208/216** | **208/216** |
| **D6S1691** | **190/201** | **178/178** | **178/210** | **201/203** | **178/201** | **190/210** | **190/203** |
| **D6S501** | **175/179** | **175/179** | **175/179** | **175/179** | **175/179** | **175/175** | **175/179** |
| **D7S513** | **196/198** | **195/208** | **186/208** | **208/208** | **196/208** | **196/208** | **198/208** |
| **D7S794** | **149/154** | **149/154** | **149/154** | **149/149** | **149/154** | **149/149** | **149/154** |
| **D8S1106** | **218/218** | **202/206** | **210/227** | **207/223** | **206/218** | **210/218** | **218/223** |
| **D9S921** | **177/181** | **177/181** | **181/185** | **173/185** | **181/181** | **177/185** | **173/177** |
| **D10S1412** | **161/164** | **161/164** | **161/161** | **161/161** | **161/164** | **161/161** | **161/161** |
| **D11S2002** | **242/250** | **230/234** | **238/242** | **238/238** | **230/242** | **242/242** | **238/250** |
| **D12S364** | **267/270** | **265/280** | **265/270** | **258/267** | **267/280** | **270/270** | **267/267** |
| **D13S765** | **217/229** | **217/233** | **225/225** | **221/225** | **217/229** | **225/229** | **225/229** |
| **D15S823** | **368/388** | **340/380** | **340/380** | **320/357** | **340/388** | **340/388** | **357/368** |
| **D18S72** | **307/335** | **307/321** | **309/321** | **317/321** | **307/307** | **321/335** | **307/321** |
| **D18S537** | **170/174** | **174/178** | **174/178** | **174/178** | **170/178** | **174/174** | **170/178** |
| **D22S685** | **337/350** | **313/357** | **329/341** | **306/338** | **313/337** | **341/350** | **306/350** |
| **DXS2506** | **273/273** | **262/273** | **262/266** | **262/273** | **262/273** | **262/262** | **262/273** |
| **MFGT21** | **122/124** | **111/128** | **114/122** | **116/116** | **124/128** | **122/122** | **116/122** |
| **MFGT22** | **94/98** | **94/111** | **88/96** | **98/106** | **94/111** | **88/98** | **94/106** |
