# Supplementary material for: Accelerated passage of gene-modified monkeys by hormone-induced precocious puberty
Source: Natl Sci Rev. 2021 May 4;8(7):nwab083. doi: 10.1093/nsr/nwab083 (PMC8310752; doi:10.1093/nsr/nwab083)
Supplement: nwab083_Supplemental_Files [file nwab083_supplemental_files.zip › Supplementary_Table_S3.docx]

| **No.PRRT2**  **F1** | **Sperm origin** | **Gender** | **Date of birth** | **Genotype** |
| --- | --- | --- | --- | --- |
| P-F1-1# | F0-P11# | **♂** | 2018/5/30 | -208bp,+5bp/WT |
| P-F1-2# | F0-P11# | **♂** | 2018/5/31 | -208bp,+5bp/WT |
| P-F1-3# | F0-P11# | **♂** | 2018/6/12 | -43bp/WT |
| P-F1-4# | F0-P11# | **♀** | 2018/7/2 | -43bp/WT |

**Supplementary Table S3. Summary of PRRT2 F1 monkeys**
