# Supplementary material for: Accelerated passage of gene-modified monkeys by hormone-induced precocious puberty
Source: Natl Sci Rev. 2021 May 4;8(7):nwab083. doi: 10.1093/nsr/nwab083 (PMC8310752; doi:10.1093/nsr/nwab083)
Supplement: nwab083_Supplemental_Files [file nwab083_supplemental_files.zip › supplementary-methods.docx]

**Methods**

**Animal ethics statement**

The use and care of animals complied with the guideline of the Animal Advisory Committee at the Shanghai Institute of Biological Science, CAS/ Center for Excellence in Brain Science and Intelligence Technology, CAS. The ethic application entitled “Reproductive physiology of cynomolgus monkey and establishment transgenic monkey” #ER-SIBS-221106P, were approved by Shanghai Institute of Biological Science, Chinese Academy of Sciences. The ethic application entitled “Monkey passage acceleration study” ION-2018003, were approved by Center for Excellence in Brain Science and Intelligence Technology, Chinese Academy of Sciences.

**Hormone injection**

Human FSH (3IU/kg/day) and testosterone (testosterone enanthate, 125mg/week) were subcutaneously injected into the experimental monkeys. The FSH is from Merck Serono (Gonal-f) and diluted in commercial buffer. The testosterone enanthate (CAS number: 315-37-7) is purchased from sinopharm group. The power of testosterone enanthate is diluted in corn oil, filtered by 0.22µm filter and injected using a 1ml syringe.

**Sperm collection**

Monkey semen was collected by penile electro-ejaculation. Ejaculated semen was liqueﬁed at 37°C for at least 15 minutes. Then the liquid sperm was washed twice with TH3 medium and the upper swimming sperm were used for ICSI.

**Oocytes collection, ICSI, and embryo transfer**

All the monkey assisted reproductive technology was guided as previous report [2]. The oocytes were collected by laparoscopy. Briefly, cycling females were twice daily injected with25 IU of recombinant human FSH (rhFSH) for 8 d. Then 1000-1500 IU of human chorionic gonadotropin (hCG) were injected on day 9. Thirty-six hours after the hCG injection, the female was anesthetized for oocytes recovery by laparoscopy. The collected oocytes were cultured in pre-equilibrated maturation medium. For ICSI, a single picked sperm was aspirated tail first and injected into the ooplasm using a piezo-actuated micromanipulator. After ICSI, embryos were cultured in pre-equilibrated hamster embryo culture medium 9 (HECM9) at 37°C under 5% CO_2_, and high-quality zygotes were selected for embryo transfer to menstruation-synchronized recipients on the next day. The pregnancies of recipients were confirmed by B ultrasonography examination 30 days after embryo transfer.

**Histological analysis**

Histological analyses were performed as follow protocol. Briefly, testes were fixed in the Bouin’s solution (Sigma, HT10132) for 16 hours, followed by dehydrating with ethanol. After that, the tissues were embedded in paraffin, cut into 5μm-thick sections and mounted on glass slides. Afterwards, the slides were immerged into xylene for dewaxing and rehydrated with gradual concentrations of ethanol. And then the sections were stained with the hematoxylin and eosin (Sangon Biotech, E607318). Whereafter, the sections were dehydrated though 95% alcohol, absolute alcohol and cleared in xylene. Finally, the slides were encapsulated with the neutral balsam mounting medium. Images were captured with an Olympus microscope system (Olympus, IX81).

**Testicular volume measurement**

For monkey testicular volume calculation, the length of long axis and short axis of monkey testis was measured every month. The volume of monkey testis was calculated as previous report [8].

**Sperm quality analysis**

Semen quality analysis was performed using the CASA system (Sperm Class Analyzer, SCA 5.2.0.1 MICROPTIC S.L., Barcelona, Spain) in the 12 samples. The CASA settings were followed according to the manufacturer's instructions. Briefly, a 5µL aliquot of semen sample was placed in the Makler chamber. At least 500 sperm were counted with CASA to evaluate the sperm concentration, sperm motility, and sperm motion variables, including curvilinear velocity (VCL), straight line velocity (VSL), average path velocity (VAP), linearity (LIN=VSL/VCL), straightness (STR=VSL/VAP), wobble (WOB=VAP/VCL), amplitude of lateral head displacement (ALH), and beat cross frequency (BCF).

**Short tandem repeats (STR) analysis**

Locus-specific primers each containing a fluorescent dye (FAM/HEX/[TMR](http://baike.baidu.com/subview/1171925/1171925.htm)) were used for PCR amplification in batches. FAM, HEX or TMR-labeled STR amplicons were diluted and mixed with internal size standard ROX500 and deionized formamide, followed by capillary electrophoresis on ABI PRISM 3730 genetic analyzer to obtain raw data. Sequenator-genetated raw data were analyzed with the program Gene Marker 2.2.0, which produces wave plots, Excel documents (including information such as size and genotype), and DNA profiles.

**DNA methylation analysis**

DNA quantification and qualification for bisulfite-sequencing (BS-Seq). Genomic DNA degradation and contamination was monitored on agarose gels. DNA purity was checked using the NanoPhotometer^®^spectrophotometer (IMPLEN, CA, USA). DNA concentration was measured using Qubit^®^DNA Assay Kit in Qubit® 2.0 Flurometer (Life Technologies, CA, USA).

Library preparation and quantification. A total amount of 5.2 microgram genomic DNA spiked with 26 ng lambda DNA were fragmented by sonication to 200-300bp with Covaris S220, followed by end repair and adenylation. Cytosine-methylated barcodes were ligated to sonicated DNA as per manufacturer’s instructions. Then these DNA fragments were treated twice with bisulfite using EZ DNA Methylation-GoldTMKit (Zymo Research). And the resulting single-strand DNA fragments were PCR amplificated using KAPA HiFi HotStart Uracil + ReadyMix (2X). Library concentration was quantified by Qubit^®^ 2.0 Flurometer (Life Technologies, CA, USA) and quantitative PCR, and the insert size was checked on Agilent Bioanalyzer 2100 system.

Clustering and sequencing (Novogene Experimental Department). The clustering of the index-coded samples was performed on a cBot Cluster Generation System using TruSeq PE Cluster Kit v3-cBot-HS (Illumia) according to the manufacturer’s instructions. After cluster generation, the library preparations were sequenced on an Illumina Hiseq 2000/2500 platform and 100 bp/50bp single-end reads were generated. Image analysis and base calling were performed with the standard Illumina pipeline, and finally 100bp paired-end reads were generated.

Quality control. Read sequences produced by the Illumina pipeline in FastQ format were first pre-processed through in-house perl scripts. Firstly, as a subset of reads contained all of part of the 3’adapter oligonucleotide sequence, every read was scanned for the adapter sequence, and if detected the read was filtered out. Secondly, since some reads had unknown base in their sequences, the percentage of Ns in each read was calculated, and if the percentage of Ns was larger than 10% the read was removed. Thirdly, reads with low quality (PHRED score <= 5, and percentage of the low qualitified bases >= 50%) were trimmed. At the same time, Q20, Q30 and GC content of the data were calculated. The remaining reads that passed the filters were called as clean reads and all of the subsequent analyses were based on them.

Reads mapping to the reference genome. Bismark software (version 0.12.5; Krueger F, 2011) was used to perform alignments of bisulfite-treated reads to a reference genome with the default parameters. The reference genome was firstly transformed into bisulfite-converted version (C-to-T and G-to-A converted) and then indexed using bowtie2 (Langmead B, 2012). Sequence reads were also transformed into fully bisulfite-converted versions (C-to-T and G-to-A converted) before they are aligned to similarly converted versions of the genome in a directional manner. Sequence reads that produce a unique best alignment from the two alignment processes (original top and bottom strand) are then compared to the normal genomic sequence and the methylation state of all cytosine positions in the read is inferred. The same reads that aligned to the same regions of genome were regarded as duplicated ones. The sequencing depth and coverage were summarized using deduplicated reads. The results of methylation extractor were transformed into bigWig format for visualization using IGV browser. The sodium bisulfite non-coversion rate was calculated as the percentage of cytosines sequenced at cytosine reference positions in the lambda genome.

Estimating methylation level. To identify the methylation site, we modeled the sum***s^+^_i,j_*** of methylated counts as a binomial (Bin) random variable with methylation rate ***r _i,j_***

***s^+^_i,j_****_~_* ***_Bin_* _(_*s^+^_i,j_*_+_*s^-^_i,j,_ r _i,j_*_)_**

We employed a sliding-window approach, which is conceptually similar toapproaches that have been used for bulk BS-Seq (<http://www.bioconductor.org/packages/2.13/bioc/html/bsseq.html)>. With window size w = 3,000 bp and step size 600 bp (Smallwood S, 2014), the sum of methylated and unmethylated read counts in each window were calculated. Methylation level (ML) for each C site shows the fraction of methylated Cs, and is defined as:

$$ML(C)=\frac{reads(mC)}{reads(mC)+ reads(C)}$$

Calculated ML was further corrected with the bisulfite non-conversion rate according to previous studies (Lister et al. 2013).M Given the bisulfite non-conversion rate r, the corrected ML was estimated as:

$$\mathrm{ML}(correct)=\frac{ML-r}{1-r}$$

**Genotype analysis**

Monkey skin tissues were excised and genomic DNA was extracted using TIANamp Genomic DNA kit (TIANGEN, Beijing, China). Genome around the targeted region was amplified with corresponding primer pairs (primer sets 5’-CAGACCAGGGGTCTAGGCT-3’ and 5’-GCATGATTACTCCTGGGGCT-3’ to amplify exon 2-targeted sequence; primer sets 5’- ACATGTGTGTCACAGCCTCG-3’ and 5’- TCAGGCTCCCTTGGTCCTTA-3’ to amplify exon 3-targeted sequence) using Extaq polymerase. PCR products were either subjected to Surveyor cleavage assay using Surveyor Mutation Detection Kit (Integrated DNA Technologies, Coralville, USA) following the manufacturer’s protocol or cloned into pGEM-T easy vectors (Promega, Madison, USA) for Sanger sequencing.
