# Supplementary figures and images for: Accelerated passage of gene-modified monkeys by hormone-induced precocious puberty
Source: Natl Sci Rev. 2021 May 4;8(7):nwab083. doi: 10.1093/nsr/nwab083 (PMC8310752; doi:10.1093/nsr/nwab083)

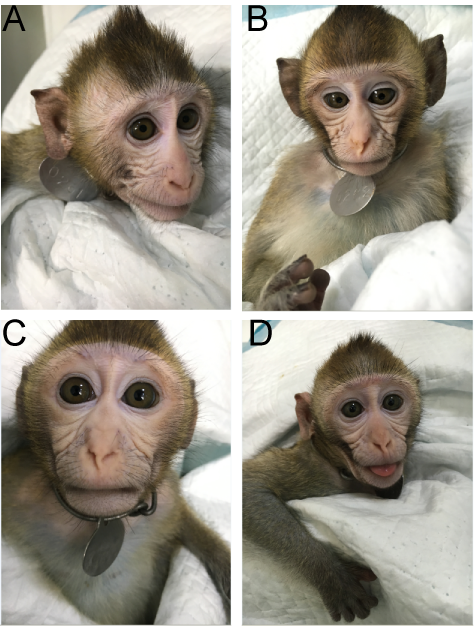

Supplement: nwab083_Supplemental_Files [file nwab083_supplemental_files.zip › Supplementary_Figure S4.tif]

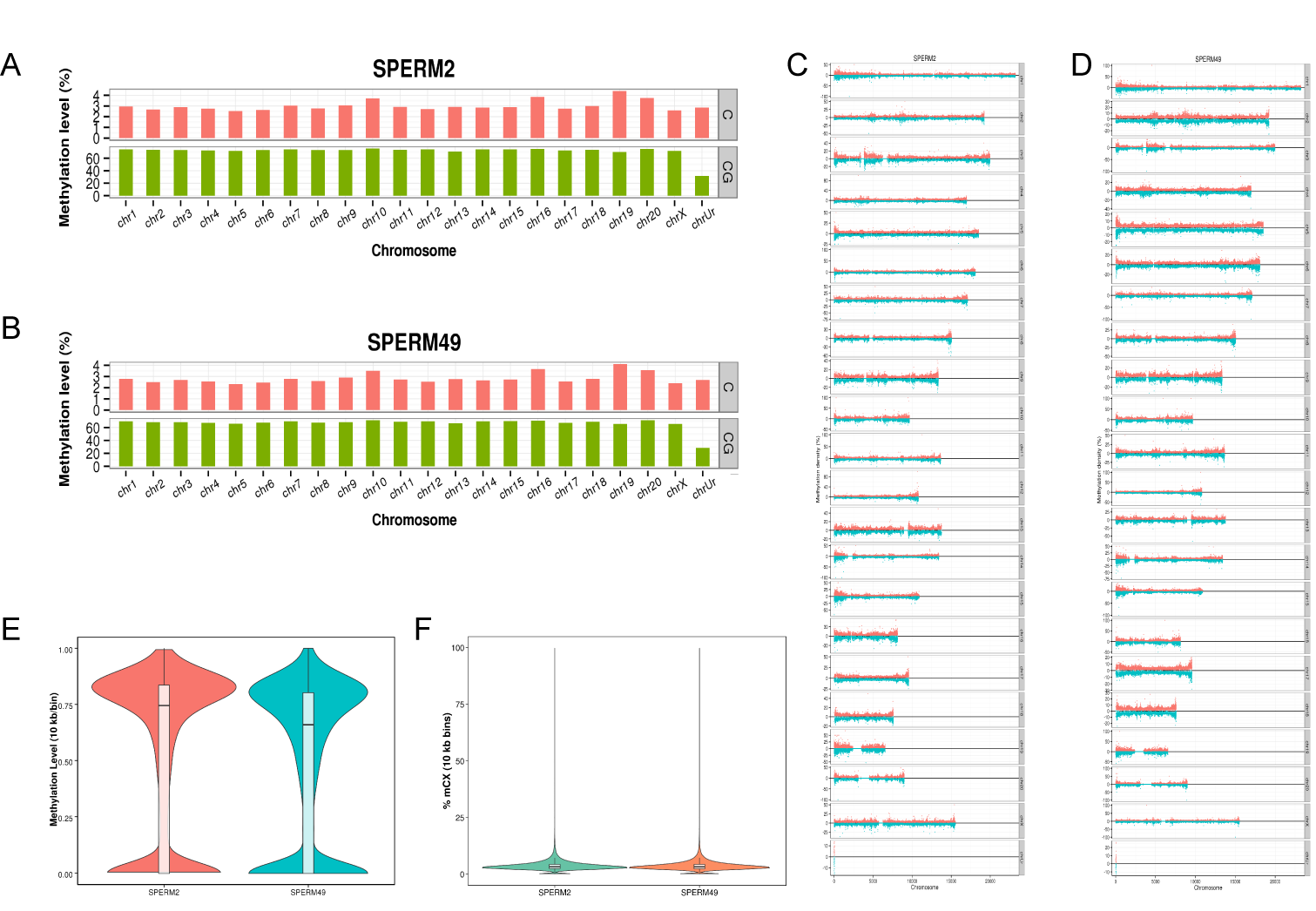

Supplement: nwab083_Supplemental_Files [file nwab083_supplemental_files.zip › Supplementary_Figure_S1.tif]

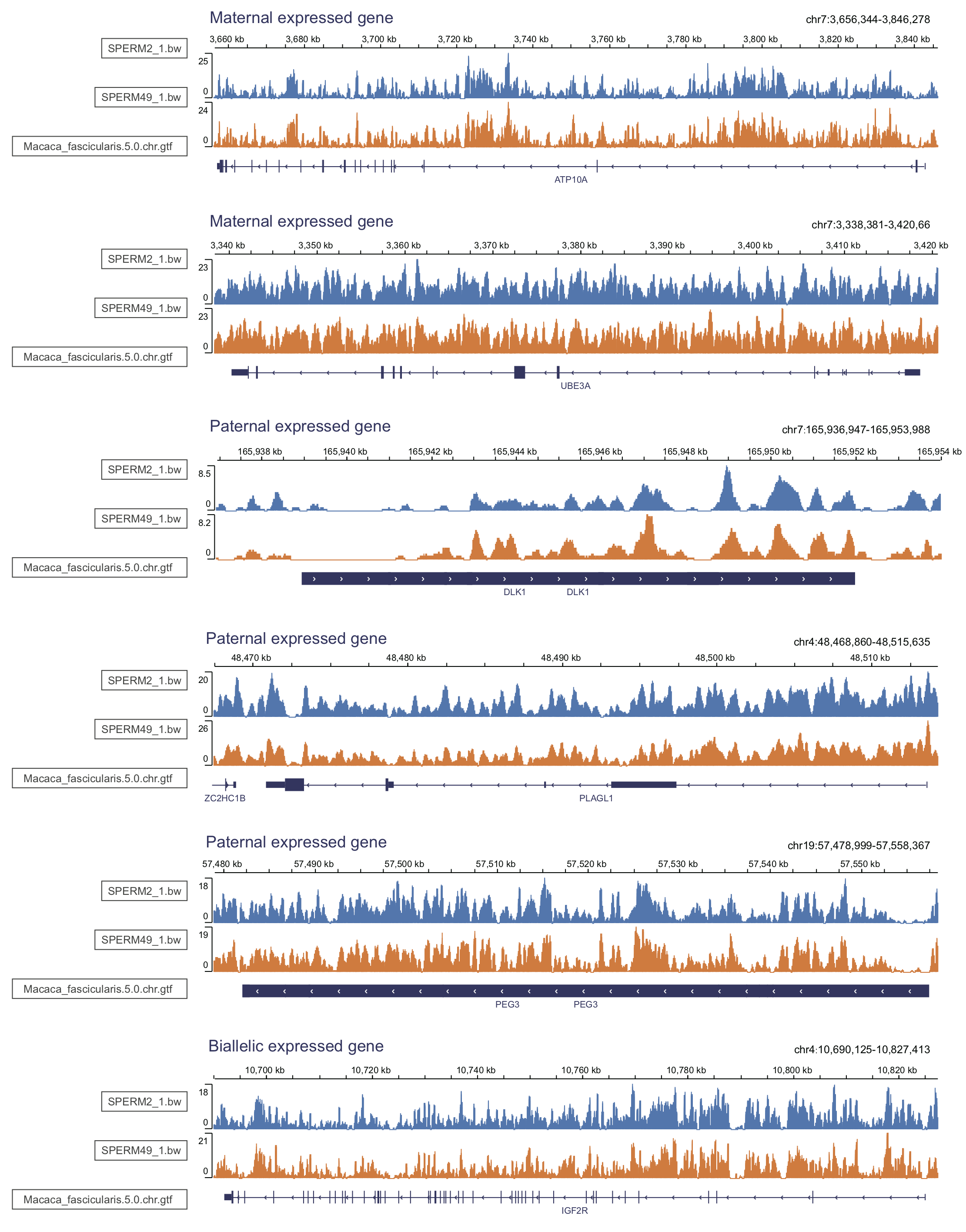

Supplement: nwab083_Supplemental_Files [file nwab083_supplemental_files.zip › Supplementary_Figure_S2.tif]

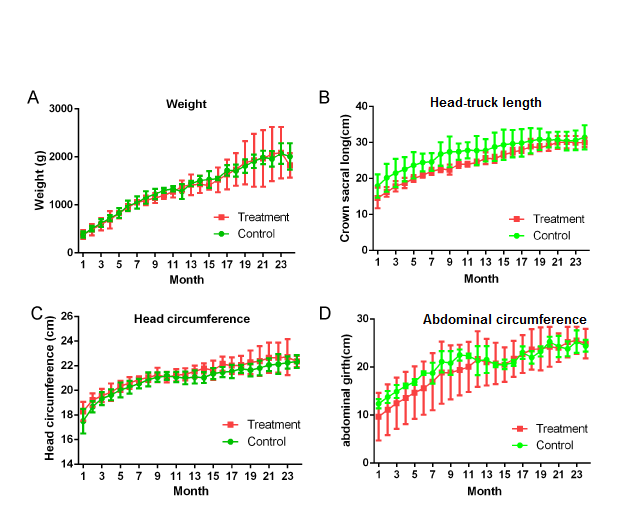

Supplement: nwab083_Supplemental_Files [file nwab083_supplemental_files.zip › Supplementary_Figure_S3.tif]

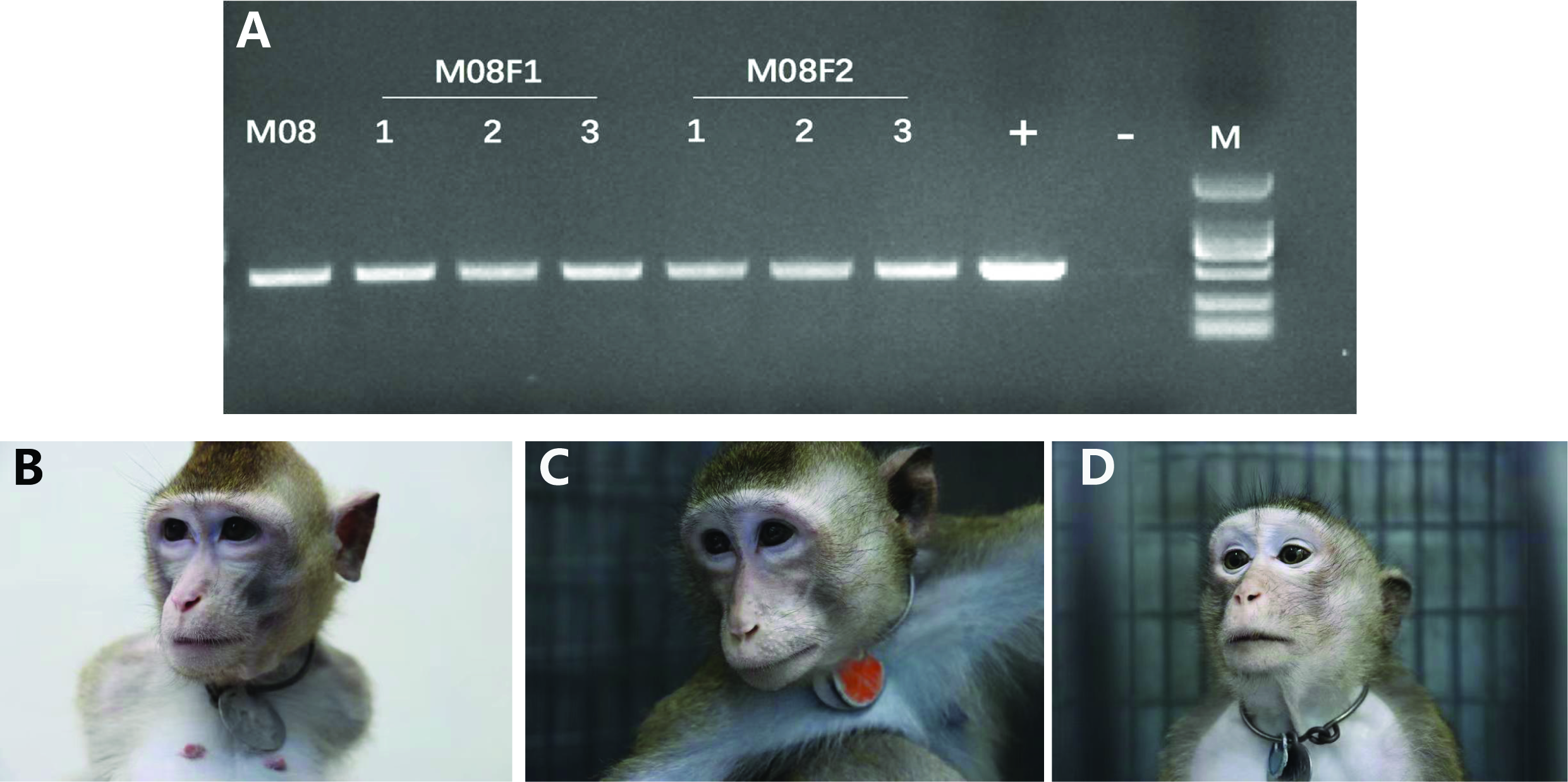

Supplement: nwab083_Supplemental_Files [file nwab083_supplemental_files.zip › Supplementary_Figure_S5.tif]
